# Hess Lancaster Screen Test with Eye Tracker: An Objective Method for the Measurement of Binocular Gaze Direction

**DOI:** 10.3390/life13030668

**Published:** 2023-02-28

**Authors:** Elvira Orduna-Hospital, Luz Maurain-Orera, Carmen Lopez-de-la-Fuente, Ana Sanchez-Cano

**Affiliations:** Department of Applied Physics, University of Zaragoza, 50009 Zaragoza, Spain

**Keywords:** Hess Lancaster screen test, eye tracker, eye movement conjugacy, eye movements, gaze positions, prismatic deviation

## Abstract

Background: To objectively measure with an eye tracker both eye movement conjugacy and gaze direction in different positions when performing the Hess Lancaster Screen Test (HLST) in a sample of control subjects without binocular dysfunction and compare the results with those of the traditional subjective HLST performance. Methods: The sample was selected avoiding subjects who suffered from suppression of one of the two eyes, visual acuity less than 20/25 on the Snellen chart in each eye, strabismus, or any symptom of binocular dysfunction that could alter the results. While performing the HLST, the examiner wrote down each of the points on a template in a traditional way while the eye tracker (Tobii Pro Fusion, Tobii AB, Danderyd, Sweden), placed in front of the subject, took objective measurements of the position of both eyes at each point. Of the 29 subjects recruited in this study, 13 subjects between 18 and 27 years old underwent the complete optometric examination and the HLST wearing anaglyph glasses; meanwhile, 16 people were excluded because of binocular or accommodative dysfunctions or because they didn’t give reliable eye-tracking results. Additionally, a specific program called Etracker Parse Video (University of Zaragoza, Zaragoza, Spain) was developed to analyse the prismatic deviation between both eyes at each evaluated point. Results: Similar horizontal prismatic deviations of visual axes were obtained in the different gaze positions with the Maddox rod, the manually annotated HLST, and the eye-tracker measurement. Variations were found in the magnitude of the deviation between methods but not in the direction. On the other hand, vertical deviations were more difficult for the examiner to detect and quantify, especially those with small magnitudes; more exact values were obtained when measuring objectively with the eye tracker. Conclusion: The HLST is very useful and allows the amount of heterophoria or heterotropia to be recorded in the patient’s medical record in all the main gaze positions. This test is complementary; by itself, it is not diagnostic and does not replace a complete examination of binocular vision. The eye tracker is an objective method with which we can evaluate the HLST in patients with no binocular problems, obtaining more accurate results than when it is performed in the traditional manner.

## 1. Introduction

Ocular motility increases the effective visual field, both monocular and binocular, allowing the visual system to keep the images of both eyes simultaneously focused on both foveas, directing the visual axes so that fusion and stereopsis occur, and avoiding diplopia [1]. Approximately 70% of the population exhibits phoria, which is a latent (hidden) deviation of the visual axes of the eyes, manifested in the absence of all stimuli to fusion [2,3,4]. However, other conditions, such as tropia, could be present; strabismus describes the imbalance of the muscles responsible for eye movements, resulting in manifest eye misalignment, with a prevalence of 4% [5]. Measuring ocular alignment and motility is essential for the diagnosis and successful treatment of both tropia and phoria with diplopia symptomatology, if possible, in the different diagnostic gaze positions. In the above-described conditions, the ocular deviation measurement method must be easy and quick to perform, reproducible under the same conditions, and comparable with the results of previous and subsequent examinations.

The different techniques for assessing ocular deviation can be divided into objective methods without the need for a response from the patient, such as light-reflex tests, in which the corneal reflections indicate the deviation, and the prism-cover test, in which compensatory eye movements are used to estimate and measure the deviation. There are also subjective methods involving the collaboration of the patient, such as the Maddox rod test, in which two objects have to be visually superimposed by the subject [6]. However, these methods are partly inaccurate because they depend on the level of experience of the examiner, who may miss very small movements, and all of them require the active cooperation of the patient to be performed [7,8]. Currently, several variants of subjective screening tests are used clinically to measure and document ocular deviations [9]. The Hess [10,11] and Lancaster [12,13] screen tests both use red–green glasses to break binocular fusion and allow an evaluation of the ocular deviation pattern in nine gaze positions at 0° and 15° deviation from the centre position, while the cover tests only assess deviations in the central gaze. In both, the targets are visible to the fixating eye, while the pointer with which the patient is to superimpose the targets is visible to the tested eye. Gaps between the target and cursor quantify the deviation angle [9]. The Harms tangent screen [14] is a similar test that serves to quantify the horizontal, vertical, and torsional components of ocular misalignment. In contrast to the Hess Lancaster screen test (HLST), for the Harms test, the head, rather than the eyes, is rotated into nine different positions to achieve an eccentric gaze.

Even with good patient cooperation, these tests have some limitations and are relatively accurate because of their high level of user interaction and lack of objectivity, as the patient must indicate the position of the light shown on the screen. In addition, the dependence on external influences such as lighting conditions as well as the inability to measure latent deviations in some patients with visual suppression or abnormal retinal correspondence are present. Some computerized versions have been developed, but they still have the same patient requirements and limitations, and the results are not always fully comparable [15,16]. On the other hand, an accurate and objective method using binocular dual search coils in a three-field magnetic system has been developed [17,18], which is semi-invasive, as it involves placing silicone rings on the patient’s cornea. It is also expensive and time-consuming for daily clinical practice. There are other alternatives using eye tracking in combination with virtual reality head-mounted displays [19] or portable strabismus video goggles, which include a head-fixed laser target display and liquid crystal display (LCD) shutters for binocular dissociation; the performance of the strabismus video goggles has been compared with the standard Hess screen test [20]. Several video-based infrared eye-tracking devices exist, but options for commercially available video recording systems suitable for binocularity assessment are scarce [21,22]. There are also very recent studies carried out with eye tracking, assessing ocular following responses in children [23] or other binocular vision and stereopsis tests [24,25]. Recent scientific evidence shows how eye tracker devices reduce the need for user interactions by directly and automatically measuring where the user is looking at. In summary, there has been a continuing search for alternative, objective, and noninvasive techniques for recording visual axis deviation, since most video recording systems are not totally adapted to binocularity measurements in daily clinical practice.

The main objective of this work is to objectively measure with an eye tracker both eye movement conjugacy and gaze direction in different positions when performing the HLST in a sample of control subjects without binocular dysfunction and to compare the results with those of the traditional subjective HLST performance.

## 2. Materials and Methods

### 2.1. Sample Description

This prospective study was approved by the Comité de Ética de la Investigación de la Comunidad de Aragón (CEICA) (reference PI21-074), and the conduct of the study adhered to the tenets of the Declaration of Helsinki. After an explanation of the nature and possible consequences of the study, written informed consent was obtained from all participants before the examination.

In this study, 29 subjects between 18 and 27 years of age underwent a complete optometric examination in which refractive error, accommodation, vergences, oculomotricity, and sensory status were very strictly evaluated. The exclusion criteria for participation in the study were suppression of one of the two eyes with the Worth test, best corrected visual acuity (BCVA) lower than 20/25 on the Snellen chart in each eye, strabismus, or any binocular dysfunction that could alter the results, or having symptoms compatible with the suspicion that the participant was suffering from dysfunction (Table S1 of Supplementary Material). It was intended that the subjects should have optometric values within the normal range for their age, and we were particularly strict with that criterion because it is conceivable that in patients with binocular problems such as strabismus or high phorias there could be a more significant number of data losses in this test. Finally, the sample included in the study was made up of 13 participants without binocular dysfunctions.

### 2.2. Experimental Protocol

The optometric tests performed in the preliminary examination evaluated the alignment of the visual axes, specifically the dissociated horizontal and vertical phoria, and fusion, both in distance vision and in intermediate vision (1 metre from each of the subjects). To assess phoria, the cover test and the Maddox rod test were performed at the central gaze position. The Worth test was used to assess both central and peripheral fusion in each of the subjects and to avoid suppressions that would prevent the HLST from being performed.

**Figure 1:**
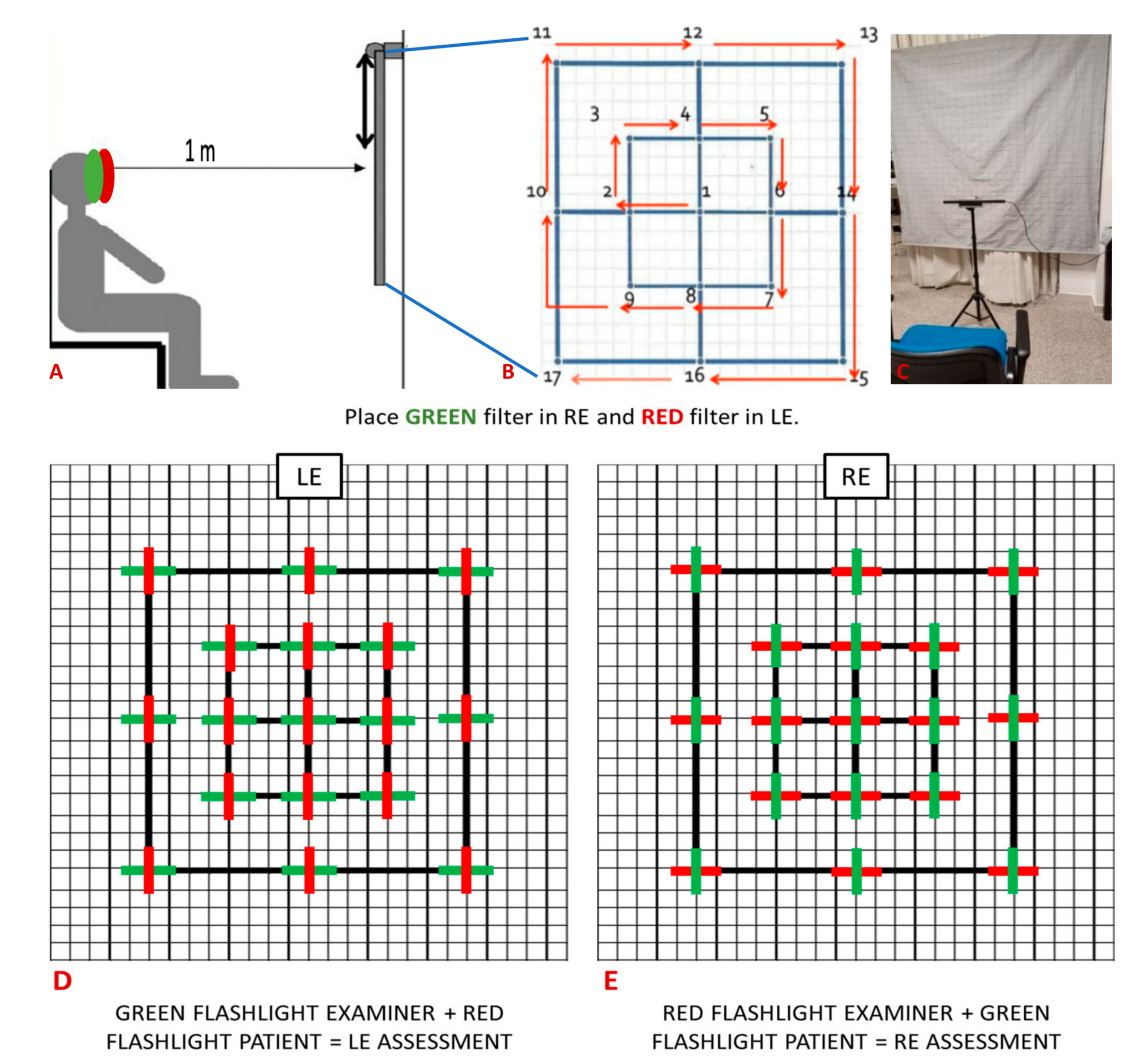
(**A**) Representation of the patient wearing glasses with a red–green filter sitting 1 m from the Hess Lancaster Screen. (**B**) Representation of the 17 points to be evaluated and the order followed during the test. (**C**) Real photo of the experiment, with the chair placed 1 metre from the screen and the eye tracker placed on a magnetized tripod 60 cm in front of the patient. (**D**,**E**) Template in which the results obtained when the test is carried out subjectively are recorded. This example simulates the results of a patient without ocular deviations. (**D**) Evaluation of a left eye (LE). (**E**) Evaluation of a right eye (RE).

Twenty-nine participants underwent the optometric evaluation and the HLST. Before the HLST and in front of the subject (Figure 1), the eye tracker was placed objectively to record the position of the eyes during the procedure, and simultaneously, the subjective results were documented by the same examiner following the traditional protocol. The eye-tracking device used in this study was the Tobii Pro Fusion eye tracker (Tobii AB, Danderyd, Sweden) with a dual-camera system and two pupil-tracking modes (bright and dark pupil) with dimensions of 374 × 18 × 13.7 mm and a capture speed of 250 Hz. To record the experiment, a camera equipped with a microphone (model AMDIS01B, Conceptronic, Dortmund, Germany) was also needed, which was directly connected to the laptop on which the Tobii Pro Fusion eye-tracker programs were installed; the eye-tracker Manager (Tobii AB, Danderyd, Sweden) for device selection and the Tobii Pro Lab (Tobii AB, Danderyd, Sweden) for calibration of the subjects in each reading were installed. The recordings and their subsequent segmentation were performed on this laptop.

To perform the HLST, the patient was placed 1 metre from the grid, sitting at the height of the central point (point 1, Figure 1B), with the head still while wearing red–green filters. One Foster flashlight was used because it projected a red-light stripe (which was seen by the eye that wore the red filter) and a green-light stripe (seen by the eye that wore the green filter). The participants were only allowed to move their eyes, and the Foster flashlight was provided at any time, depending on the eye to be evaluated.

The explorer projected the light horizontally, and the participant projected his light vertically, forming a cross and following an established order (Figure 1B,D,E), until the completion of the 17 points to be evaluated.

In this study, the patient wore custom-made anaglyph glasses with the green filter on the right eye (RE) and the red filter on the left eye (LE) perfectly matching the lights projected by Foster’s flashlights. These filters were lighter than the standard filters to make it easier for the eye tracker to detect the pupils, avoiding losing pupil position during the probe. The template where we recorded the results manually was properly modified for these characteristics.

The protocol began with the examination of the LE. The explorer projected the green light horizontally, and the patient projected the red light vertically. The patient tried to form the cross following the order shown in Figure 1B while the examiner marked each of the 17 points. The explorer had to point to where the patient placed the red-light stripe with respect to the examiner’s green stripe on the left grid of the template (corresponding to the LE) (Figure 1D).

Next, the RE evaluation was performed. The patient placed the green light vertically, and the examiner placed the red light horizontally. The participant made the same crosses as those for the LE in the same order. This time, the examiner pointed to the grid on the right side of the template, which corresponds to the RE (Figure 1E).

**Figure 2:**
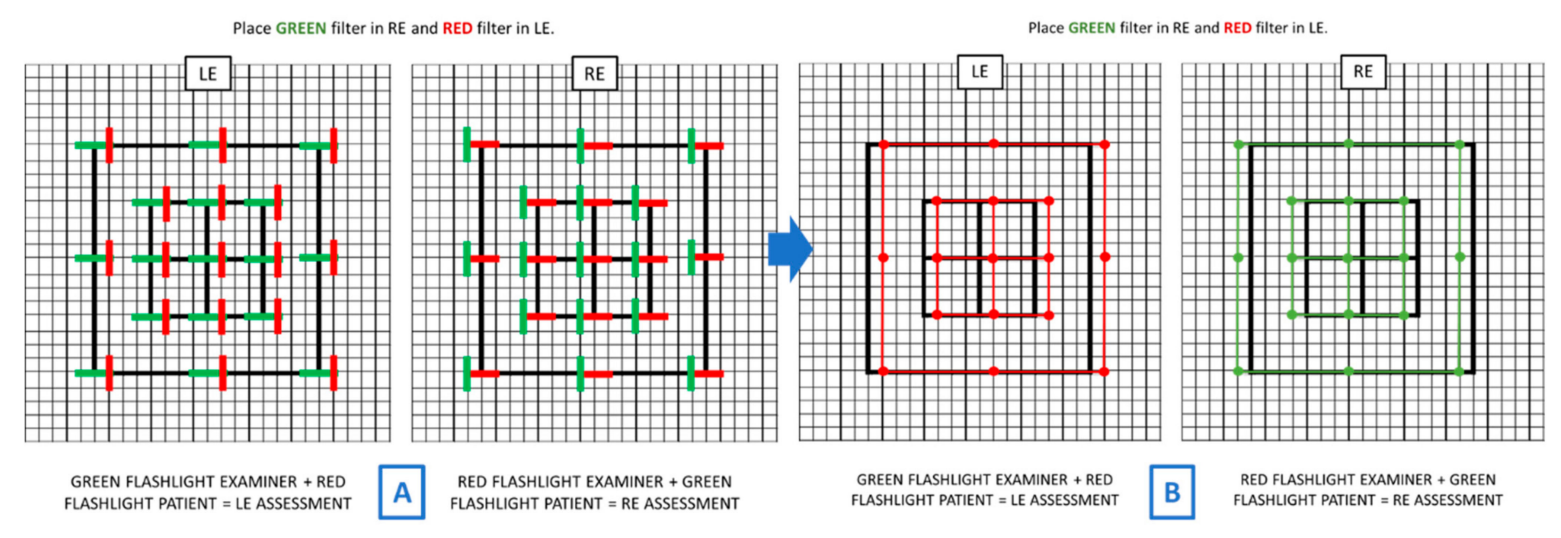
(**A**) Endophoria of 5 ∆ represented as the patient positioning his flashlight in relation to the examiner’s. (**B**) Points represented and joined by the examiner on the template where the patient placed the flashlight in relation to his own, simulating endophoria of 5 ∆.

If the lights were crossed correctly when performing the test, the patient did not have any type of deviation (orthophoria). If the light stripes remained separated, either horizontally or vertically, although the patient saw them crossed, the patient had dissociated phoria (tropias were excluded), and if the light stripes remained inclined, the patient had cyclodeviation [12,26,27]. Considering that each square of the grid measured 5 cm on each side and that the test was carried out at a distance of 1 metre, each square of deviation was equivalent to 5 ∆ [28] (Figure 2). The eye had to rotate at positions 2, 4, 6, and 8 an angle of 11.31°, at positions 3, 5, 7, and 9 an angle of 15.79°, at positions 10, 12, 14, and 16 an angle of 21.80°, and at positions 11, 13, 15, and 17 an angle of 29.50°. During the calibration, the eye tracker was able to cover a total horizontal and vertical (to the right, left, up, and down) angle of 26.57° (10 frames) and a diagonal angle of 35.26°. The extreme diagonals were almost outside the maximum angle that the eye tracker can measure (35°), and for this reason it was decided to take the 9 central points for this study. We wanted to obtain accurate data.

In addition, while the HLST was being performed, the eye tracker was placed 60 cm in front of the patient to detect and monitor eye movements, thus obtaining both subjective (marked by the examiner on the template) and objective (detected by the eye tracker) measurements.

The study elements were arranged so that the measurements were taken with the magnetized tripod placed on marks on the floor so that all measurements were taken at the same distance from both the grid screen and the patient and could be compared. The eye tracker was placed on this tripod and connected to a laptop. There was a chair 100 cm from the HLST and 60 cm from the eye tracker. Placed in the back of the room, behind the patient, a camera connected to the laptop recorded the test to allow us to represent the location of the fixations on the video after finishing the test.

The selection of the Tobii Pro Fusion device and its configuration were performed using the eye-tracker Manager, and the calibrations were improved using the Tobii Pro Lab program. Objectively, the camera recorded the positions on the grid screen where the subject was looking on the HLS; the positions should have been coincident with those marked in the Tobii Pro Lab program.

A final calibration was then performed with the patient wearing the red–green filters. If one of the two eyes was not detected by the eye tracker, the patient was excluded. Therefore, during the calibration, the patient was asked to look at the points indicated by the examiner in the same order in all cases.

After calibration, the test was performed under scotopic lighting conditions (<1 lux), evaluating the LE first followed by the RE. At the same time, the results were noted in the template by the examiner, and the data were recorded objectively by the eye tracker. It was very important to ensure that the eye tracker did not lose the ability to detect any of the eyes when performing the test; otherwise, the test was not valid.

### 2.3. Data Collection

**Figure 3:**
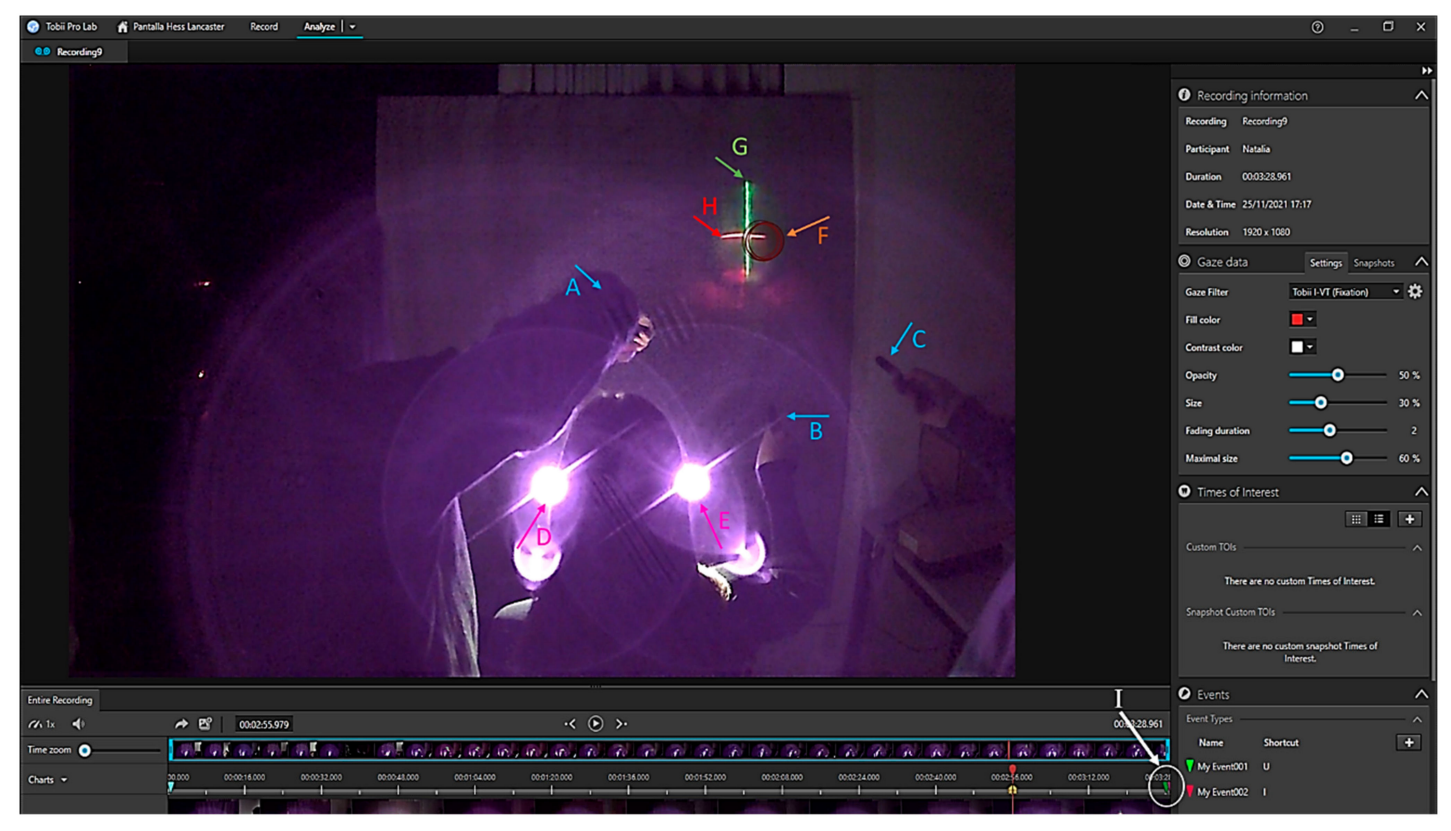
Screenshot of the Tobii Pro Lab program during the analysis of the recording and segmentation of the two events. (A) The auxiliary examiner illuminates the scene slightly with a flashlight to be able to capture the image, since all tests were performed in scotopic lighting conditions. (B) The participant holding the green Foster’s flashlight vertically for evaluation of the right eye (RE). (C) The examiner holding the red Foster’s flashlight horizontally for RE evaluation. (D,E) Infrared lights emitted by the eye tracker to detect the patient’s eyes by the reflections in their pupils. (F) Red circle indicating where the eye tracker detects that the patient is looking. (G) Vertical green light projected by the participant. (H) Horizontal red light projected by the examiner. (I) Marking the end of an event and the beginning of the next with the corresponding triangles.

Once the test had been carried out on all the participants, all the recordings were reviewed in Tobii Pro Lab and segmented into two “events”, a first part where the exploration of the LE began and ended (My Event001 U in green) and a second part where the RE was tested (My Event002 I in red). The “event” is what the program calls the selected time intervals between two marks (Figure 3). To delimit the “event”, two marks are set manually by the examiner, one at the beginning and one at the end of the evaluation for each eye. They are performed by pressing the letter “u” on the keyboard to delimit the LE and by pressing the letter “i” to delimit the RE evaluation.

**Figure 4:**
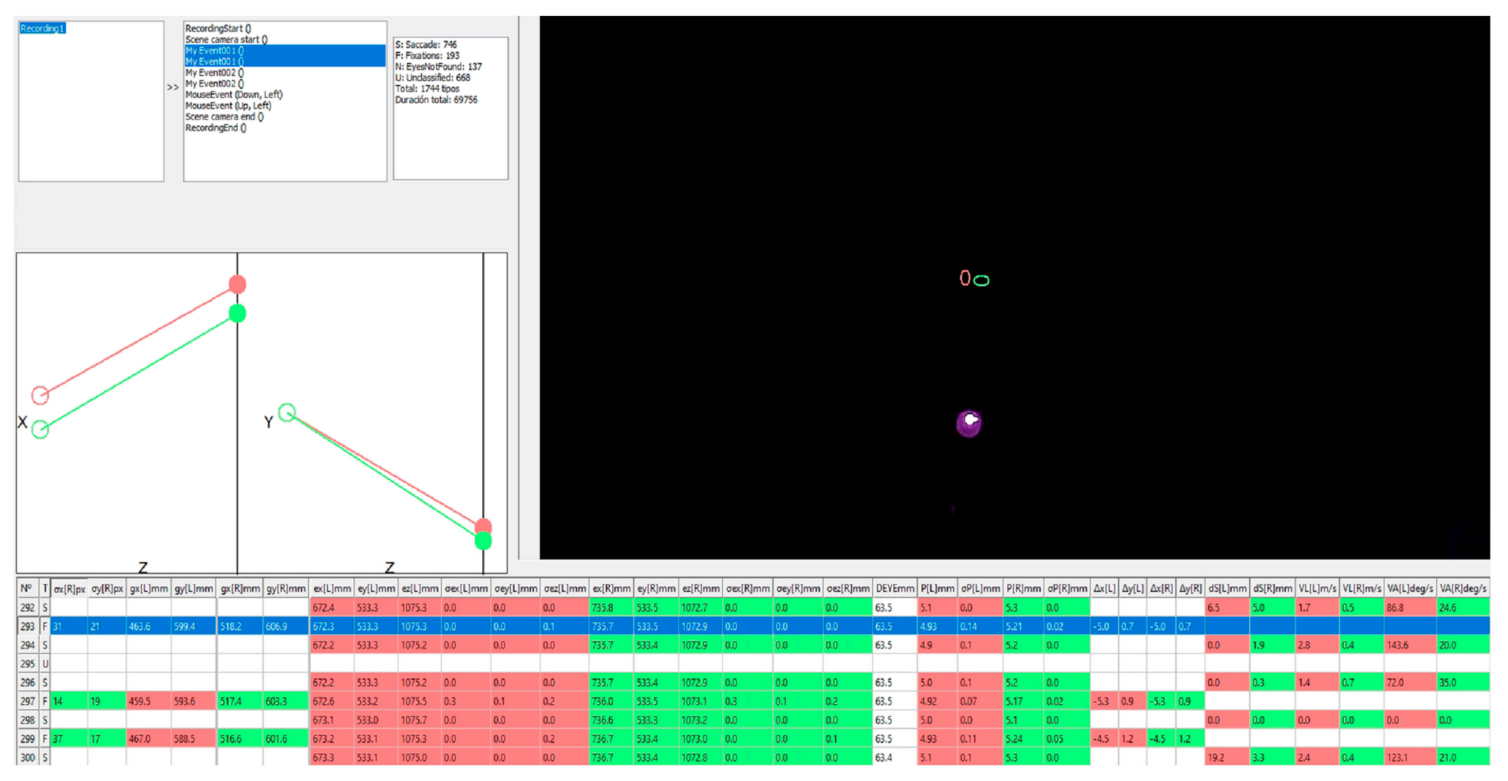
Analysis of the left eye (LE) “event” with the Etracker Parse Video program. In the box on the left is the complete recording of a participant to be analysed, and in the box next to it is the recording segmented into two events (My Event001 for the LE evaluation and My Event002 for the right eye (RE) evaluation). Just below, the patient’s eyes are represented in the horizontal (x) and vertical (y) planes and where their visual axes meet. On the left is a video image with two fixations, the green one belonging to the RE and the red one to the LE. In the grid below with red (RE) and green (LE) backgrounds, the values of the variables of interest are analysed. The line marked in blue corresponds to the two fixations (RE and LE) represented in the video image.

**Figure 5:**
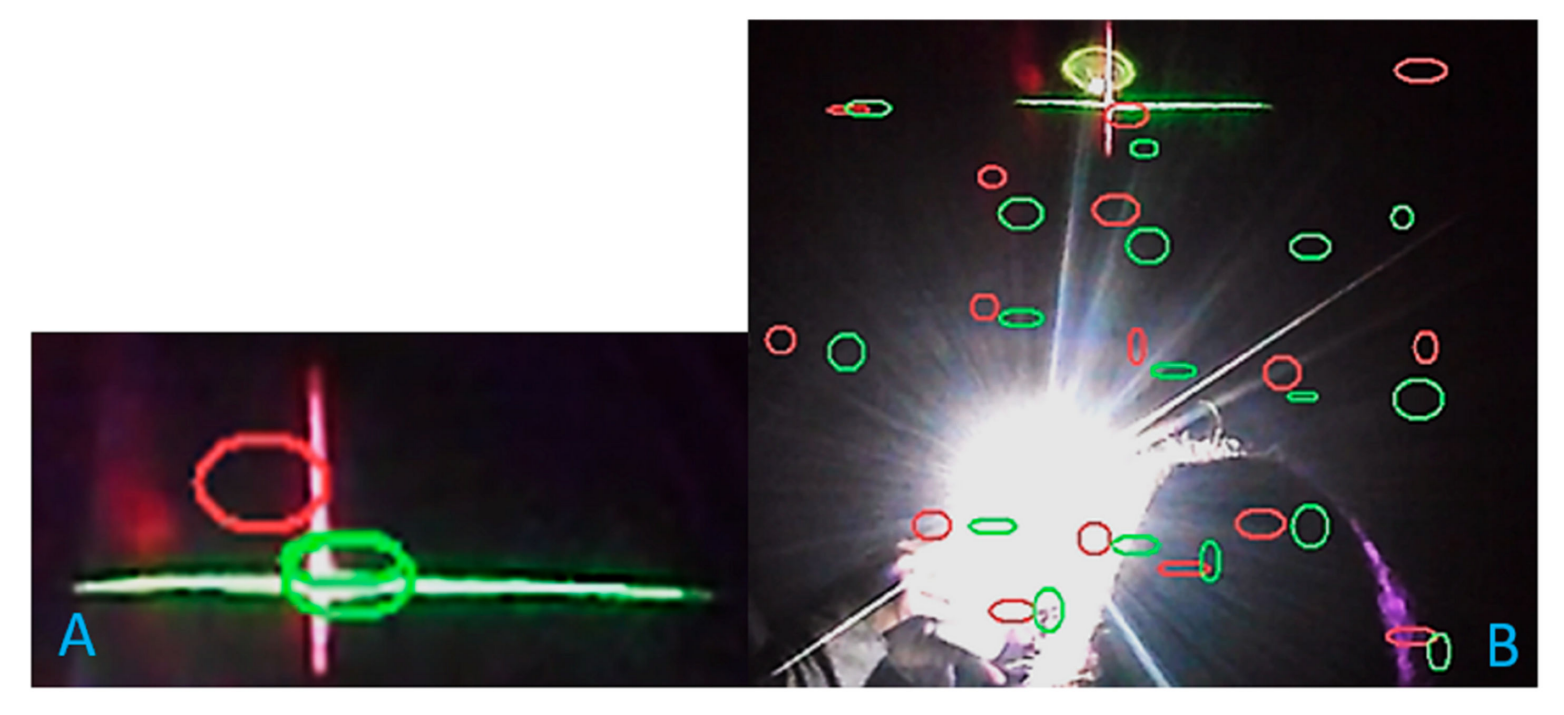
(**A**) Fixation on the central point of the Hess Lancaster screen, where the difference between the subjective examination (crossing of the red (LE) and green (RE) flashlight beams) and the objective examination by the eye tracker (the red (LE) and green (RE) ellipses) can be seen. Image from the Etracker Parse Video program. (**B**) Representation with the Etracker Parse Video program where the eye tracker detects that each of the two eyes of the participant is looking at each of the 17 gaze positions for the evaluation of the left eye.

The data from each segmented recording were then individually and automatically exported from Tobii Pro Lab to Excel (Microsoft^®^ Office Excel 2011, Microsoft Corporation, Redmond, WA, USA). To manage the amount of data that Tobii Pro Lab exported, a specific program called Etracker Parse Video (University of Zaragoza, Zaragoza, Spain) was developed. The exported Excel data provided by the device were imported to analyse the deviation in prismatic diopters that existed between the LE fixation with respect to the RE at each of the points explored during the test and were re-exported from the Etracker Parse Video program to Excel to create our graphs, according to the coordinates (x, y, z) taken by the eye tracker of the position of each eye with respect to the grid screen (Figure 4 and Figure 5), and grouped into a much more manageable database, with the variables of all of the recordings combined for statistical analysis.

### 2.4. Statistical Analysis

The measurements of the variables to be studied were recorded in an Excel database, with which the figures in the results section were made, according to the coordinates (x, y, z) of the positions of each eye at each point explored. In addition, the prismatic deviations were analysed at each point studied and for each patient, depending on whether they had been obtained manually or automatically, using the Statistical Package for the Social Sciences (SPSS 20, SPSS Inc., IBM Corporation, Somers, NY, USA). The normal distribution of the values was examined with the Kolmogorov–Smirnov test. Since the values had a normal distribution, a paired sample *t*-test was used to evaluate whether there were differences between the measurement methods of the same subject for each point, both horizontally and vertically, and their correlation. A *p* value < 0.05 was considered statistically significant.

## 3. Results

To carry out this study the test was performed on 29 subjects; they were asked to bring contact lenses whenever possible to avoid confusion with the pupillary reflex and prismatic effects produced by the ophthalmic lenses of their glasses in the different gaze positions. Then, 16 people were discarded owing to binocular dysfunctions (11) or problems with the eye tracker in detecting the pupillary reflex (5) and therefore not obtaining valuable measurements (Table S1 of Supplementary Material). Finally, 13 subjects with a mean age of 21.92 ± 2.02 years and a mean refractive error of −2.83 ± 1.91 D, of whom 7 wore contact lenses, completed the test, including 8 women and 5 men. The recording of the test with the eye tracker lasted for 3.78 ± 0.56 min (mean ± standard deviation).

### Subjective Method vs. Objective Method Results

**Figure 6:**
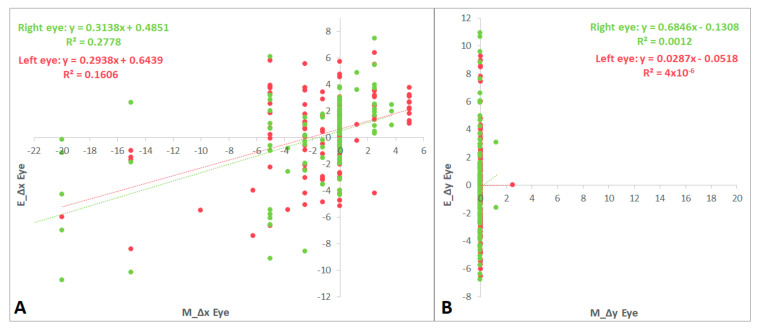
Scatter plots comparing the two HLST measurement methods (manual and with eye tracker) in the 9 points of the 13 participants (117 points), as well as the regression line calculated for both horizontal (**A**) and vertical deviation (**B**). M_∆x Eye: Manual horizontal measurements; M_∆y Eye: Manual vertical measurements, E_∆x Eye: Eye-tracker horizontal measurements; E_∆y Eye: Eye-tracker vertical measurements. In red for the left eye and in green for the right eye.

Once all the values of horizontal and vertical deviations were obtained for the nine points of the prismatic deviations when evaluating each eye in the HLST of each subject, the values were compared to determine if there were statistically significant differences between the values obtained when performing the test subjectively and objectively and if there was a correlation between the two methods. This information is shown in Table 1 and Figure 6.

Differences between the measurements and the correlation coefficient (cc) obtained when comparing the results of the horizontal (∆x) and vertical (∆y) deviations collected subjectively by the examiner versus the objective results collected by the eye tracker in a sample size of 117 measurements (9 points from 13 subjects). A value of *p* < 0.05 was considered statistically significant.

The horizontal measurements of the RE and LE collected manually and by the eye tracker were compared separately. There were statistically significant differences for both eyes (for the RE *p* < 0.001 and for the LE *p* < 0.001), so different results were obtained subjectively from objectively. A statistically significant positive correlation was seen for both eyes in horizontal measurements (LE: cc = 0.401, *p* < 0.001 and RE: cc = 0.527, *p* < 0.001), which indicated that although there were statistically significant differences between the value of the measurements, if the manual measurement was an esophoria, the eye tracker also measured an esophoria, and if the examiner detected an exophoria, it was also detected by the eye tracker. There were differences in the absolute value of the subjective and objective measurements but not in the sign or direction of the deviation.

This comparison between subjective and objective measurements was also made for the vertical deviation and for each eye separately. There were no statistically significant differences between the values of prismatic deviations measured by both methods (LE: *p* = 0.815 and RE: *p* = 0.650), but there was no correlation between them (LE: cc = 0.002, *p* = 0.983 and RE: cc = 0.034, *p* = 0.716). This indicated that the vertical deviation could not be detected by the examiner but could be measured by the eye tracker or that the examiner detected an RE hyperphoria that the eye tracker detected as RE hypophoria (as occurred in Subject 3 in the Supplementary Material).

**Scheme 1:**
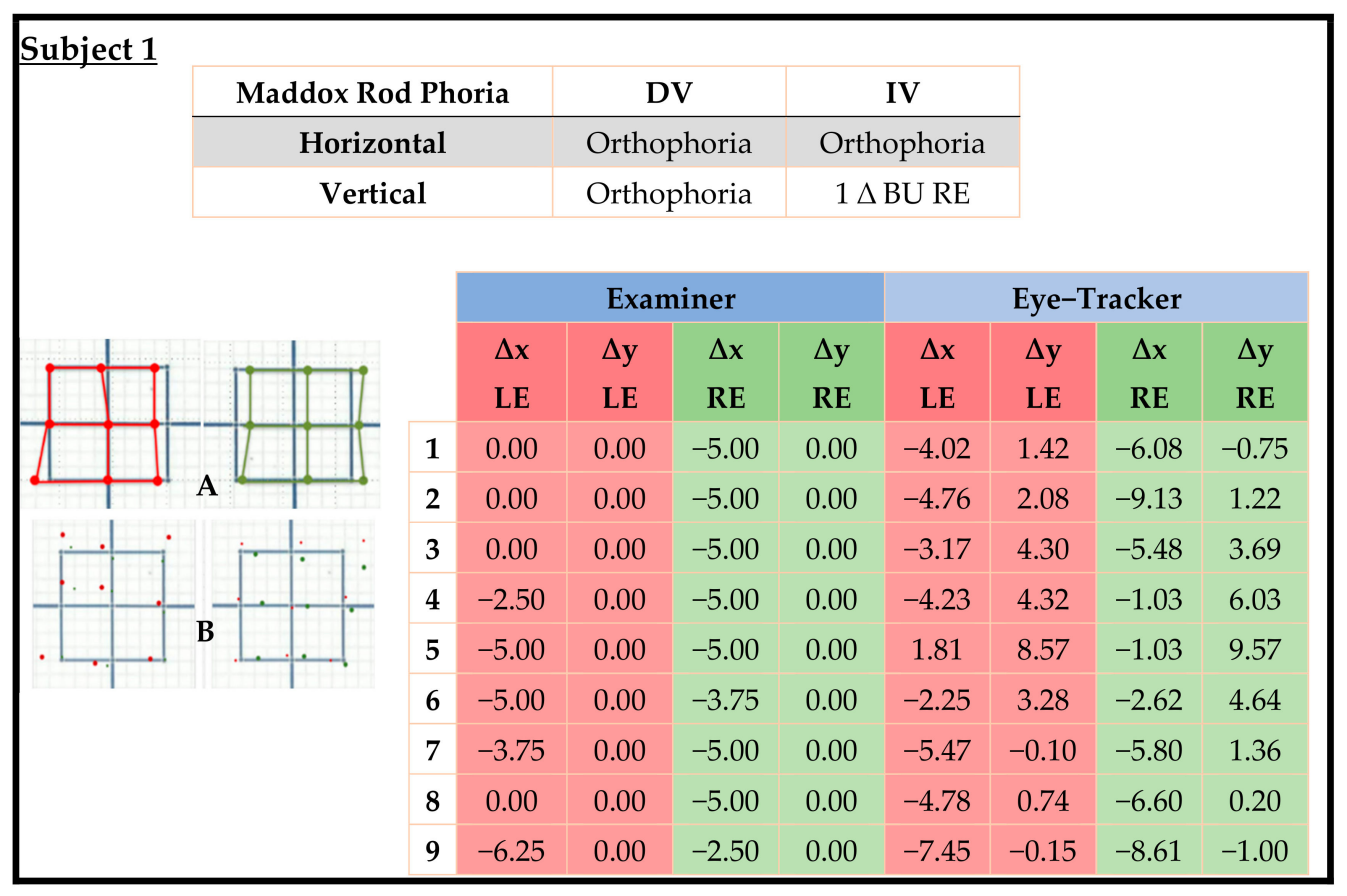
Data of a study subject. At the top is a table with the subjective data of the values of horizontal and vertical phoria measured with the Maddox rod test for both distance vision (DV) and intermediate vision (IV). On the bottom right is another table with the data, both objective (fixations collected by the eye tracker at the 9 central points of the Hess Lancaster screen test) and subjective (estimated deviations after manual data collection by the explorer and reflected in the Hess Lancaster test template), including horizontal (∆x) and vertical (∆y) deviations for each eye. On the bottom left is the representation of the Hess Lancaster screen obtained manually (red for the LE and green for the RE (illustration A)) and the representation of the points obtained with the eye tracker (red points for the LE and green for the RE (illustration B).

An example of the data collected, both subjectively by the examiner (measurement of the horizontal and vertical phoria in both distance and intermediate vision with the Maddox rod and the Hess Lancaster screen) and objectively (the Hess Lancaster screen with the eye tracker), is represented in Scheme 1. For the analysis of the results of the Hess Lancaster screen, nine central points were considered (Figure 1), since the measurements taken by the eye tracker were much more precise at this area. The peripheral points 10 to 17 showed poor eye-tracking precision; there were a number of detection losses by the eye tracker in many patients, and these were not analysed.

It should be noted that the program we have created to analyse the eye-tracker data uses the prismatic deviations, taking as a reference how the LE deviates from the RE, which is why we always show some small points and other large ones (the large ones corresponding to the eye that we are evaluating), so that it can be seen that the numerical deviation that we show in the table is always from one point to the other (illustrations A and B of Scheme 1). Thus, an exophoria will have a negative sign (−), while an esophoria will have a positive (+) sign. In the case of vertical deviations, RE hyperphoria will be a (+) sign, and RE hypophoria will be a (−) sign.

The discussion, interpretation, and detailed analysis of the deviations found in three subjects are attached as Supplementary Material, as well as the results of the thirteen participants (Table S2 of the Supplementary Material).

## 4. Discussion

In this work, we wanted to first develop a protocol based on objective measurements of the HLST by eye tracker and, second, validate it by comparing the results with those of the standard subjective procedure. This study was performed on 29 subjects; 11 of them were discarded owing to binocular or accommodative dysfunctions and 5 of them due to problems with the eye tracker in detecting the pupillary reflex (5) and therefore not obtaining valuable measurements. Our study showed that when analysing the nine central points of the HLST subjectively (measurements taken manually by the examiner) and objectively (measurements taken by the eye tracker), the same or very similar results were found horizontally but were very different vertically, since it depends greatly on the subjectivity of the examiner performing the test, who in this study was always the same examiner.

Our findings in terms of horizontal deviation were similar among all the test modalities carried out (Maddox rod test, manual HLST, and measurement with eye tracker), but variations were found in the magnitude of the deviation, although not in direction (i.e., if there was an esophoria, it was detected as an esophoria by all three modalities). No modality is diagnostic by itself, but all of them are complementary to the rest of the optometric tests that would be performed during an optometric examination. On the other hand, vertical deviations were more difficult for the examiner to detect and quantify, resulting in different values when measured objectively with the eye tracker, especially in regard to small-magnitude values, as was the case in this study.

Currently, thanks to technological advances, efforts are being made to improve the reliability and reproducibility of this test by trying to make it more objective. It should be noted that when performing the HLST subjectively, the examiner notes where the patient places the flashlight vertically relative to the examiner’s horizontal flashlight. By contrast, when monitoring the HLST with an eye tracker, the device records where the pupillary reflex is in each patient’s eye and determines where on the screen each eye is fixating. Then, our custom-made program calculates the prismatic deviation between pupillary reflexes. For the examiner, determining and recording on the template exactly where the patient places the flashlight is imprecise, since each square of 5 cm equals 5 ∆. Therefore, when the test is carried out manually, there may be errors that depend not only on the examiner’s subjectivity but also on the patient’s hand–eye coordination when aiming the flashlight, as well as the tremor of the patient’s hand. These factors disappear when performing the test with the eye tracker, since no subjective factor is involved, but rather the device monitors the pupillary reflex and calculates which part of the screen each patient’s eye is fixing on without the need to evaluate the distance between the lights. In this way, the test is much more exact and precise and allows us to determine very small deviation values less than 1 ∆ that are subjectively undetectable, as was observed with the vertical deviations.

There are very few studies available in the literature on the utility of the HLST in oculomotor palsies [29,30], with only one [31] where superior oblique palsy was assessed in downgaze with respect to the PGP and one by Armesto et al. [32] performing the HLST with the head straight and tilted to both sides to investigate the eye movements during Bielschowsky head-tilt testing. Weber et al. [20] designed video goggles with the target projection fixed on the head, and a distant white wall was sufficient for projection without the need to use a chin rest. Head-free recording also allows for Bielschowsky head-tilt testing for diagnosing patients with fourth nerve palsy [18]; in our study, we had the advantage of having the head free and being able to monitor the eye movements and inclination seen from the front with the eye tracker.

Traditional screen tests to map ocular deviations, such as the Hess screen test [10], the Lancaster red–green test [12], or the Harms tangent screen test [14,33], are measurement methods widely used for strabismus diagnosis and follow-up [6]. However, they all require the understanding and cooperation of the patient in the test and experienced examiners specialized in the subject, since they must correctly record the subject’s responses on paper. In addition, these tests are very time-consuming, and any failure or doubt about the answer implies its re-evaluation. In our method, because the measurements are not examiner dependent, a trained technician can operate the eye tracker and collect the data for later evaluation by the treating physician. In addition, Thorisdottir et al. [34] showed that the digital KM screen test is less time-consuming and that the majority of patients preferred it to other conventional techniques. On the other hand, it is worth noting that the historical gold standard is oculography based on eye movement measurements with scleral search coils [17,18,21], but this test is semi-invasive, cumbersome, and only possible in specialized laboratories. An advantage of objective eye movement devices is the possibility of measuring even subjects with visual suppression, such as congenital comitant strabismus [35]. Mehringer et al. [19] developed a new version of the Hess screen test that combines the advantages of objective measurement by eye tracking with the environmental consistency offered by virtual reality (VR) head-mounted displays (HMDs). However, they did not implement an individual calibration procedure, whereas this was done in our study.

In patients with a significant refractive correction or one in which prisms are incorporated, contact lenses are helpful. Since most tests require the patient to keep their head still, looking through a spectacle lens at an angle will create distortion or a prismatic effect if the target is viewed off of the optical centre [36]. 

There are several limitations to this study. First, there is always the possibility of bias when examiners cannot be masked to the results, although all patients were examined by the same orthoptist. The data from the eye tracker were recorded automatically on a computer, which eliminates the variability that may be introduced by the observer when marking the results on the separate paper template; this may lead to a higher degree of variability. Second, our study cohort was small, since the eye tracker did not correctly detect the eyes of all the participants when carrying out the test, and we had to exclude five participants. Third, only participants with low phorias were included in the study, so, due to the lack of range and continuity in values reported, the statistical analysis is limited, and the results cannot be extrapolated to other population groups; to improve this point the described method should be corroborated with other ocular conditions that were considered as exclusion criteria. Despite these limitations, our study indicates that the HLST monitored with an eye tracker appears to be an objective, accurate, and time-saving complementary method for the quantification and follow-up of ocular deviations in different gaze positions. A future study including patients with diagnosed strabismus could provide more insight into the validity of the measurements.

## 5. Conclusions

In conclusion, the HLST is very useful and allows the amount of heterophoria or heterotropia to be recorded in the patient’s medical record in all the main gaze positions. This test is complementary; by itself, it is not diagnostic and does not replace a complete examination of binocular vision. The eye tracker is an objective method with which we can evaluate the HLST in patients with no binocular problems, obtaining more accurate results than when it is performed in the traditional manner.

## Figures and Tables

**Table 1:** Differences between the horizontal (∆x) and vertical (∆y) deviations in the subjective versus the objective examinations.

Examiner vs. Eye-Tracker Values	Differences (*p*)	Correlation Coefficient (cc) (*p*)
∆x LE	<0.001	0.401 (<0.001)
∆y LE	0.815	0.002 (0.983)
∆x RE	<0.001	0.527 (<0.001)
∆y RE	0.650	0.034 (0.716)

Differences between the measurements and the correlation coefficient (cc) obtained when comparing the results of the horizontal (∆x) and vertical (∆y) deviations collected subjectively by the examiner versus the objective results collected by the eye tracker in a sample size of 117 measurements (9 points from 13 subjects). A value of *p* < 0.05 was considered statistically significant.

## Data Availability

The data sets of the current study are available from the corresponding author upon reasonable request.

## Supplementary Materials

The following supporting information can be downloaded at: https://www.mdpi.com/article/10.3390/life13030668/s1, Table S1: Optometric values of the 13 included study subjects and of the 16 excluded subjects with their diagnosis; Table S2: Data of the 13 study subjects.
