# Supplementary material for: Hess Lancaster Screen Test with Eye Tracker: An Objective Method for the Measurement of Binocular Gaze Direction
Source: Life (Basel). 2023 Feb 28;13(3):668. doi: 10.3390/life13030668 (PMC10054291; doi:10.3390/life13030668)
Supplement: Supplementary file 1 [file life-13-00668-s001.zip › life-2199824-supplementary.pdf]

life

MDPI

Article

Hess Lancaster Screen Test with Eye Tracker: An Objective Method for the Measurement of Binocular Gaze Direction

Elvira Orduna-Hospital *, Luz Maurain-Orera, Carmen Lopez-de-la-Fuente and Ana Sanchez-Cano

Department of Applied Physics, University of Zaragoza, 50009 Zaragoza, Spain
* Correspondence: eordunahospital@unizar.es

SUPPLEMENTARY MATERIAL

S3. Results

The 29 participants with all the optometric values of the different tests measured and, in the case of those excluded, their diagnosis of binocular dysfunction or the loss of one or both eyes by the eye-tracker are presented in this supplementary material as Table S1.

Citation: Orduna-Hospital, E.; Maurain-Orera, L.; Lopez-de-la-Fuente, C.; Sanchez-Cano, A. Hess Lancaster Screen Test with Eye Tracker: An Objective Method for the Measurement of Binocular Gaze Direction. *Life* **2023**, *13*, 668. https://doi.org/10.3390/life13030668

Academic Editor: Akio Oishi

Received: 18 January 2023
Revised: 24 February 2023
Accepted: 27 February 2023
Published: 28 February 2023

Copyright: © 2023 by the authors. Licensee MDPI, Basel, Switzerland. This article is an open access article distributed under the terms and conditions of the Creative Commons Attribution (CC BY) license (https://creativecommons.org/licenses/by/4.0/).

Table S1: Optometric values of the 13 included study subjects and of the 16 excluded subjects with their diagnosis.

nº	Age (years)	Horizontal Phoria DV	Horizontal Phoria IV	Vertical Phoria DV	Vertical Phoria IV	AC/A	NPC (cm)	Worth DV	Worth IV	Stereopsis	NFV DV (BL/BR/RE)			NFV IV (BL/BR/RE)			PFV DV (BL/BR/RE)			PFV IV (BL/BR/RE)			VF 3/12 IV cpm	NRA (D)	PRA (D)	AA (D) RE	AA (D) LE	BAF IV cpm	MAF IV cpm RE/LE	Included/ Excluded	Excluded why?
**1**	22	0	0	0	1	5.9	5/8	4	4	16''	X	10	6	X	18	12	6	10	6	12	20	14	14	3.50	4.25	20	20	18	18/17	Included	
**2**	20	-1	-4	0	0	4.7	5/9	4	4	50"	X	12	8	X	20	16	20	25	18	35	40	35	18	2.25	2.75	25	25	13	15/16	Included	
**3**	22	3	2	2	1	5.5	7/9	4	4	25"	4	6	2	12	16	10	4	18	6	20	25	18	15	3.25	3.25	11	11	10	14/14	Included	
**4**	24	0	0	0	0	5.9	7/9	4	4	25"	X	6	2	12	18	10	2	10	2	12	18	10	19	2.5	3	25	25	17	20/19	Included	
**5**	23	0	0	0	0	5.9	4/7	4	4	25"	6	10	4	X	16	14	12	16	10	X	20	14	14	3.75	3	14	14	14	16/15	Included	
**6**	22	1	1	0	0	5.9	6/9	4	4	12.5"	4	6	1	8	12	10	8	30	6	12	30	12	12	2.75	2.25	12.5	12.5	12	16/16	Included	
**7**	21	0	0	0	0	5.9	7/9	4	4	16'	X	8	6	X	12	6	X	25	12	X	30	20	15	3	4.75	11	12	14	14/13	Included	
**8**	22	0	-1	0	0	5.5	7/9	4	4	25''	X	7	4	X	12	8	X	11	7	8	19	12	15	2.75	2.50	14	14	16	14/14	Included	
**9**	19	0	-1	0	0	5.5	6/8	4	4	12.5'	16	18	16	30	35	30	14	25	18	X	20	18	23	2.5	11.25	20	20	16	19/17	Included	
**10**	20	2	2	0	0	5.9	2/7	4	4	16	8	12	8	X	10	8	X	16	14	X	30	25	10	3.25	5.75	16.66	17	13	14/15	Included	
**11**	22	4	2	0	0	5.1	6/9	4	4	16"	X	8	4	12	16	12	10	18	12	20	30	16	17	2.75	8.25	14	14	14	15/15	Included	
**12**	21	4	2	0	0	5.1	4/8	4	4	16"	10	12	8	10	18	14	6	35	14	18	45	25	16	2.75	3.25	16	15	11	13/13	Included	
**13**	27	1	1	0	0	5.9	6/9	4	4	16"	X	8	6	8	10	6	8	16	6	14	20	14	18	2.75	3.25	14	14	10	13/14	Included	
**14**	21	3	-1	0	0	4.3	10/16	4	4	25"	6	12	6	6	12	4	6	30	12	14	20	14	14	2.75	0.75	8	8	3	8/8	Excluded	Accommodative insufficiency
**15**	20	-1	-2	0	0	5.5	7/12	4	4	50''	4	6	2	12	16	14	12	16	10	16	18	10	14	3	6.25	12.5	12.5	15	14/13	Excluded	**The eye-tracker did not find the RE**
**16**	21	0	-5	0	0	3.9	7/14	4	4	100"	8	10	6	X	16	14	4	18	8	X	10	8	12	2.75	2.25	12.5	12.5	16	16/16	Excluded	Convergence insufficiency
**17**	22	3	2	2	1	5.5	7/11	4	4	40''	6	10	8	10	12	10	25	40	25	14	35	25	18	1	0.75	11.11	11.11	22	23/22	Excluded	**The eye-tracker lost both eyes alternately**
**18**	22	-1	0	0	0	6.3	8/10	4	4	16	12	18	12	X	16	12	12	18	10	X	18	12	14	2	1	9	9	7	7/8	Excluded	Accommodative insufficiency
**19**	20	1	0	0	0	5.5	8/11	4	4	16''	12	16	10	X	18	12	12	18	8	X	18	12	14	2.75	7	14.28	14.28	12	13/12	Excluded	**The eye-tracker lost both eyes alternately**
**20**	23	1	-7	1	0	2.7	9/11	4	4	100"	6	10	2	X	14	10	4	12	2	4	12	4	17	1.25	0.25	3.85	3.85	5	5/4	Excluded	Convergence and accommodative insufficiency
**21**	24	0	-5	0	0	3.9	7/11	4	4	20"	5	12	6	X	10	4	6	10	8	X	10	6	12	0.75	2.25	20	20	4	15/15	Excluded	Convergence insufficiency
**22**	22	0	0	0	0	5.9	7/15	2	4	16"	X	8	4	X	12	10	4	18	14	16	25	16	14	2.25	2.25	14	14	14	16/15	Excluded	LE suppression with red/green filters
**23**	20	0	0	0	0	5.9	8/12	4	4	50''	X	10	8	X	8	6	X	10	6	X	8	6	0	2.25	1.25	9	9	6	5/5	Excluded	Vergence infacility and accommodative insufficiency
**24**	20	4	1	0	0	4.7	11/13	2	4	25'	6	8	4	14	25	18	4	25	12	X	4	2	18	3	5.75	16	16	5	4/3	Excluded	LE suppression with red/green filters and Accommodative infacility
**25**	18	0	0	0	0	5.9	8/16	4	4	16"	X	10	8	X	18	16	12	45	18	X	35	20	19	3.25	1.75	4	4	3	4/3	Excluded	Accommodative insufficiency
**26**	21	0	-6	0	0	3.5	9/10	4	4	63'	2	4	2	8	10	6	10	18	12	X	4	2	20	1.75	2.25	12.5	12.5	7	18/17	Excluded	Convergence insufficiency
**27**	20	-1	-1	0	0	5.9	11/13	4	4	16"	X	10	6	X	25	18	25	35	30	40	45	40	22	3.25	4.25	16.6	16.6	13	19/20	Excluded	**The eye-tracker lost both eyes alternately**
**28**	21	-1	-1	0	0	5.9	9/13	4	4	25"	X	8	4	X	16	4	X	25	18	X	30	25	17	2.5	1.75	14.25	14.25	10	13/13	Excluded	**The eye-tracker did not find the LE**
**29**	21	10	5	0	0	3.9	5/11	5	4	100"	X	4	1	X	8	2	X	10	6	X	20	14	10	2.25	1	16	16	17	14/15	Excluded	Divergence insufficiency

* AC/A, accommodative convergence to accommodation; NPC, Near point of convergence; PFV, Positive fusional vergence; NFV, negative fusional vergence; IV intermediate vision; DV, Distance vision; (BL/BR/RE), blur, break, and recovery; VF, Vergence facility with 3 base-in/12 base-out; NRA, negative relative accommodation; PRA, positive relative accommodation; AA, amplitude of accommodation; BAF, binocular accommodative facility; MAF, monocular accommodative facility

The results of the 13 participants without binocular dysfunctions are attached here as supplementary material with another two cases interpreted and discussed (Subject 3 and Subject 9).

Table S2: Data of the 13 study subjects. At the top is a table with the subjective data of the values of horizontal and vertical phoria measured with the Maddox rod test for both distance vision (DV) and intermediate vision (IV). On the bottom right is another table with the data, both objective (fixations collected by the eye-tracker at the 9 central points of the Hess Lancaster screen test) and subjective (estimated deviations after manual data collection by the explorer and reflected in the Hess Lancaster test template), including horizontal (Δx) and vertical (Δy) deviations for each eye. On the bottom left is the representation of the Hess Lancaster screen obtained manually (red for the LE and green for the RE (illustration A)) and the representation of the points obtained with the eye-tracker (red points for the LE and green for the RE (illustration B).

Subject 1

Maddox rod phoria	DV	IV
**Horizontal**	Orthophoria	Orthophoria
**Vertical**	Orthophoria	1 Δ BU RE

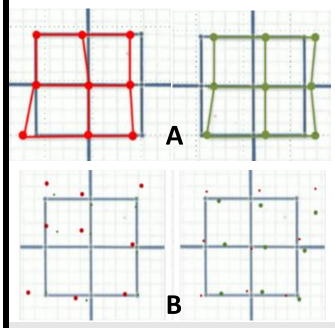

	EXAMINER				EYE-TRACKER			
	**Δx LE**	**Δy LE**	**Δx RE**	**Δy RE**	**Δx LE**	**Δy LE**	**Δx RE**	**Δy RE**
**1**	0.00	0.00	-5.00	0.00	-4.02	1.42	-6.08	-0.75
**2**	0.00	0.00	-5.00	0.00	-4.76	2.08	-9.13	1.22
**3**	0.00	0.00	-5.00	0.00	-3.17	4.30	-5.48	3.69
**4**	-2.50	0.00	-5.00	0.00	-4.23	4.32	-1.03	6.03
**5**	-5.00	0.00	-5.00	0.00	1.81	8.57	-1.03	9.57
**6**	-5.00	0.00	-3.75	0.00	-2.25	3.28	-2.62	4.64
**7**	-3.75	0.00	-5.00	0.00	-5.47	-0.10	-5.80	1.36
**8**	0.00	0.00	-5.00	0.00	-4.78	0.74	-6.60	0.20
**9**	-6.25	0.00	-2.50	0.00	-7.45	-0.15	-8.61	-1.00

Subject 2

Maddox rod phoria	DV	IV
**Horizontal**	1 Δ exophoria	4 Δ exophoria
**Vertical**	Orthophoria	Orthophoria

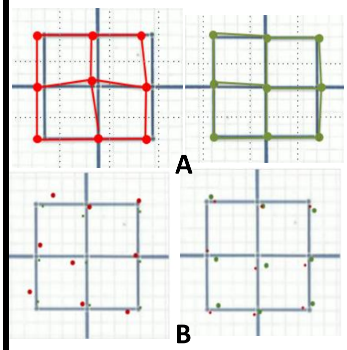

	EXAMINER				EYE-TRACKER			
	**Δx LE**	**Δy LE**	**Δx RE**	**Δy RE**	**Δx LE**	**Δy LE**	**Δx RE**	**Δy RE**
**1**	-2.50	2.50	0.00	0.00	-5.12	0.02	-4.35	-0.98
**2**	-2.50	0.00	0.00	1.25	1.72	4.78	-3.17	3.07
**3**	-2.50	0.00	0.00	1.25	3.75	4.10	1.16	-1.62
**4**	-2.50	0.00	0.00	0.00	2.46	3.70	-0.63	0.15
**5**	-5.00	0.00	0.00	0.00	5.76	0.04	-1.77	1.63
**6**	-2.50	0.00	-2.50	0.00	-2.44	2.12	-2.52	-2.13
**7**	-2.50	0.00	-1.25	0.00	5.53	0.03	-3.56	-1.65
**8**	0.00	0.00	0.00	0.00	-2.67	0.38	-1.97	-1.13
**9**	-2.50	0.00	0.00	0.00	-3.05	3.35	-3.11	-2.61

Subject 3

Maddox rod phoria	DV	IV
**Horizontal**	3 Δ esophoria	2 Δ esophoria
**Vertical**	2 Δ BD RE	1 Δ BD RE

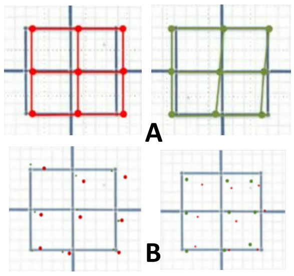

	EXAMINER				EYE-TRACKER			
	**Δx LE**	**Δy LE**	**Δx RE**	**Δy RE**	**Δx LE**	**Δy LE**	**Δx RE**	**Δy RE**
1	2.50	0.00	1.25	0.00	1.33	-3.67	3.56	-4.31
2	2.50	0.00	2.50	0.00	3.15	-1.05	5.46	-1.67
3	2.50	0.00	2.50	0.00	6.35	-1.33	7.45	-2.25
4	2.50	0.00	0.00	0.00	3.54	-2.54	2.10	-3.47
5	2.50	0.00	0.00	0.00	5.50	-2.23	3.70	-3.70
6	2.50	0.00	1.25	0.00	1.55	0.25	4.87	1.07
7	2.50	0.00	2.50	0.00	2.35	-1.20	3.70	-2.20
8	2.50	0.00	2.50	0.00	3.01	-0.68	2.10	0.40
9	2.50	0.00	2.50	0.00	3.47	1.13	3.95	2.73

Subject 4

Maddox rod phoria	DV	IV
**Horizontal**	Orthophoria	Orthophoria
**Vertical**	Orthophoria	Orthophoria

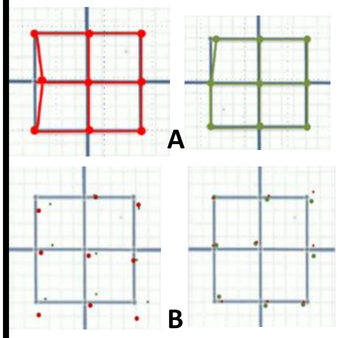

	EXAMINER				EYE-TRACKER			
	**Δx LE**	**Δy LE**	**Δx RE**	**Δy RE**	**Δx LE**	**Δy LE**	**Δx RE**	**Δy RE**
1	0.00	0.00	0.00	0.00	-2.75	-1.02	0.30	0.65
2	2.50	0.00	0.00	0.00	-4.23	-2.86	-1.70	-0.01
3	0.00	0.00	-2.50	0.00	-4.27	-2.43	-0.57	-0.23
4	0.00	0.00	0.00	0.00	0.70	-0.55	0.63	1.25
5	0.00	0.00	0.00	0.00	-0.26	1.15	0.47	2.93
6	0.00	0.00	0.00	0.00	-1.29	-0.26	-0.38	3.27
7	0.00	0.00	0.00	0.00	-5.20	-5.45	0.27	0.88
8	0.00	0.00	0.00	0.00	-1.94	-4.18	0.58	1.52
9	0.00	0.00	0.00	0.00	-3.01	-5.32	1.07	-1.40

Subject 5

Maddox rod phoria	DV	IV
**Horizontal**	Orthophoria	Orthophoria
**Vertical**	Orthophoria	Orthophoria

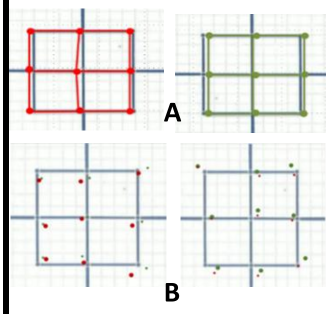

	EXAMINER				EYE-TRACKER			
	**Δx LE**	**Δy LE**	**Δx RE**	**Δy RE**	**Δx LE**	**Δy LE**	**Δx RE**	**Δy RE**
1	-2.50	0.00	-1.25	0.00	-2.10	-1.27	-0.22	-2.76
2	-2.50	0.00	-2.50	0.00	0.90	-0.68	-0.30	-1.03
3	-2.50	0.00	-2.50	0.00	-0.99	-0.87	0.15	-0.53
4	-1.25	0.00	-1.25	0.00	-3.16	-1.99	1.48	-2.08
5	-1.25	0.00	-1.25	0.00	-2.98	-2.74	1.75	-4.70
6	-1.25	0.00	-1.25	0.00	-3.23	-2.16	-0.77	-2.66
7	-1.25	0.00	-1.25	0.00	-4.89	-3.35	-3.55	-3.92
8	-1.25	0.00	-1.25	0.00	0.58	-2.78	-1.55	-2.73
9	-2.50	0.00	-2.50	0.00	1.12	-0.80	-1.97	-2.73

Subject 6

Maddox rod phoria	DV	IV
**Horizontal**	1 Δ esophoria	1 Δ esophoria
**Vertical**	Orthophoria	Orthophoria

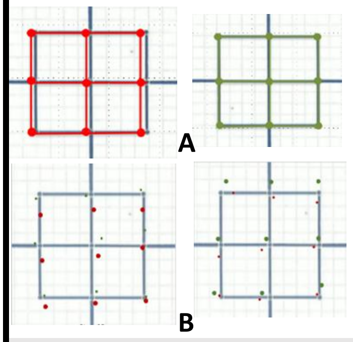

	EXAMINER				EYE-TRACKER			
	Δx LE	Δy LE	Δx RE	Δy RE	Δx LE	Δy LE	Δx RE	Δy RE
1	-1.25	0.00	0.00	0.00	-1.27	-5.70	-1.91	-3.46
2	-1.25	0.00	0.00	0.00	2.88	-4.81	0.40	-5.32
3	-1.25	0.00	0.00	0.00	1.60	-4.90	2.99	-3.47
4	-1.25	0.00	0.00	0.00	3.40	-4.27	1.08	-4.43
5	-2.50	0.00	0.00	0.00	0.65	-6.01	-0.46	-6.37
6	-2.50	0.00	0.00	0.00	-0.62	-5.41	-1.70	-2.69
7	-2.50	0.00	0.00	0.00	0.08	-1.18	-1.70	-2.69
8	-1.25	0.00	0.00	0.00	-0.51	-3.76	-1.00	-1.93
9	-1.25	0.00	0.00	0.00	0.39	-3.36	1.78	-0.70

Subject 7

Maddox rod phoria	DV	IV
**Horizontal**	Orthophoria	Orthophoria
**Vertical**	Orthophoria	Orthophoria

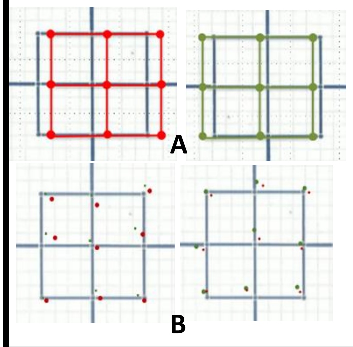

	EXAMINER				EYE-TRACKER			
	Δx LE	Δy LE	Δx RE	Δy RE	Δx LE	Δy LE	Δx RE	Δy RE
1	5.00	0.00	2.50	0.00	3.05	-2.72	1.90	-2.61
2	5.00	0.00	3.75	0.00	3.74	-1.70	2.46	-0.63
3	5.00	0.00	3.75	0.00	2.67	-2.20	1.95	-0.93
4	5.00	0.00	2.50	0.00	2.20	-3.63	2.50	-1.00
5	5.00	0.00	2.50	0.00	2.10	-2.05	1.64	-1.06
6	5.00	0.00	2.50	0.00	3.20	-2.27	0.45	-1.40
7	5.00	0.00	2.50	0.00	2.60	-2.03	0.87	-1.40
8	5.00	0.00	2.50	0.00	1.76	-3.03	0.28	-0.51
9	5.00	0.00	3.75	0.00	1.24	-0.94	0.90	0.23

Subject 8

Maddox rod phoria	DV	IV
**Horizontal**	Orthophoria	1 Δ exophoria
**Vertical**	Orthophoria	Orthophoria

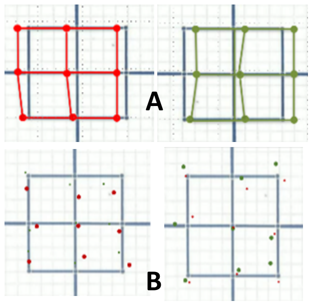

	EXAMINER				EYE-TRACKER			
	Δx LE	Δy LE	Δx RE	Δy RE	Δx LE	Δy LE	Δx RE	Δy RE
1	-5.00	0.00	-2.50	0.00	0.71	-0.98	0.10	0.30
2	-5.00	0.00	-5.00	0.00	2.53	1.45	6.05	2.05
3	-5.00	0.00	-5.00	0.00	0.20	-4.67	0.67	-2.57
4	-5.00	0.00	-5.00	0.00	3.30	-3.63	2.00	-3.22
5	-5.00	0.00	-5.00	0.00	3.70	-2.60	3.15	-5.08
6	-5.00	0.00	-5.00	0.00	3.92	-2.12	1.03	1.30
7	-5.00	0.00	-5.00	0.00	3.83	-3.77	2.80	-5.76
8	-2.50	0.00	-5.00	0.00	3.51	1.92	-0.63	-1.15
9	-2.50	0.00	-2.50	0.00	0.01	-3.09	1.48	-0.71

Subject 9

Maddox rod phoria	DV	IV
**Horizontal**	Orthophoria	1 Δ exophoria
**Vertical**	Orthophoria	Orthophoria

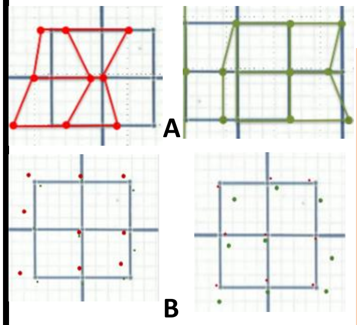

	EXAMINER				EYE-TRACKER			
	**Δx LE**	**Δy LE**	**Δx RE**	**Δy RE**	**Δx LE**	**Δy LE**	**Δx RE**	**Δy RE**
**1**	-5.00	0.00	-20.00	0.00	-0.08	2.13	-0.20	3.70
**2**	-6.25	0.00	-15.00	0.00	-4.00	8.97	-2.60	-6.80
**3**	-5.00	0.00	-20.00	0.00	-6.68	6.03	-10.78	7.62
**4**	-15.00	0.00	-20.00	0.00	-1.03	2.43	-4.30	5.90
**5**	-10.00	0.00	-20.00	0.00	-5.52	7.44	-7.00	10.65
**6**	-20.00	0.00	-15.00	0.00	-6.00	9.25	-10.18	10.93
**7**	-15.00	0.00	-22.50	0.00	-1.73	7.80	-8.53	-3.80
**8**	-15.00	0.00	-20.00	0.00	-1.53	8.47	-1.20	6.60
**9**	-15.00	0.00	-15.00	0.00	-8.45	4.25	-1.90	8.80

Subject 10

Maddox rod phoria	DV	IV
**Horizontal**	2 Δ esophoria	2 Δ esophoria
**Vertical**	Orthophoria	Orthophoria

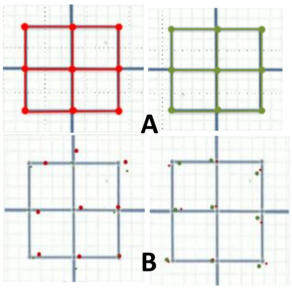

	EXAMINER				EYE-TRACKER			
	**Δx LE**	**Δy LE**	**Δx RE**	**Δy RE**	**Δx LE**	**Δy LE**	**Δx RE**	**Δy RE**
**1**	0.00	0.00	0.00	0.00	2.72	0.58	1.45	0.43
**2**	0.00	0.00	0.00	0.00	3.74	-0.58	2.16	0.40
**3**	0.00	0.00	0.00	0.00	5.69	1.11	-4.02	-0.51
**4**	0.00	0.00	0.00	0.00	1.83	3.30	2.08	-0.08
**5**	0.00	0.00	0.00	0.00	-0.59	2.43	1.08	1.22
**6**	0.00	0.00	0.00	0.00	1.53	1.60	1.33	-1.30
**7**	0.00	0.00	0.00	0.00	0.41	0.04	2.48	0.84
**8**	0.00	0.00	0.00	0.00	1.36	3.40	0.35	0.45
**9**	0.00	0.00	0.00	0.00	2.36	0.98	0.575	-0.43

Subject 11

Maddox rod phoria	DV	IV
**Horizontal**	4 Δ esophoria	2 Δ esophoria
**Vertical**	Orthophoria	Orthophoria

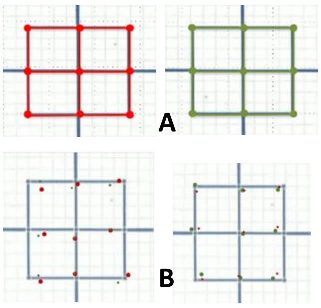

	EXAMINER				EYE-TRACKER			
	**Δx LE**	**Δy LE**	**Δx RE**	**Δy RE**	**Δx LE**	**Δy LE**	**Δx RE**	**Δy RE**
**1**	0.00	0.00	0.00	0.00	0.17	-0.25	0.27	0.71
**2**	0.00	0.00	0.00	0.00	2.93	0.60	3.77	0.47
**3**	0.00	0.00	0.00	0.00	0.78	-2.36	0.93	-2.39
**4**	0.00	0.00	0.00	0.00	3.03	1.19	0.65	0.08
**5**	0.00	0.00	0.00	0.00	4.54	1.33	0.80	1.30
**6**	0.00	0.00	0.00	0.00	0.21	-0.33	1.18	1.50
**7**	0.00	0.00	0.00	0.00	4.72	2.30	2.00	-2.58
**8**	0.00	0.00	0.00	0.00	0.17	-0.25	-0.85	0.68
**9**	0.00	0.00	0.00	0.00	0.90	-1.82	0.30	-1.76

Subject 12

Maddox rod phoria	DV	IV
**Horizontal**	4 Δ esophoria	2 Δ esophoria
**Vertical**	Orthophoria	Orthophoria

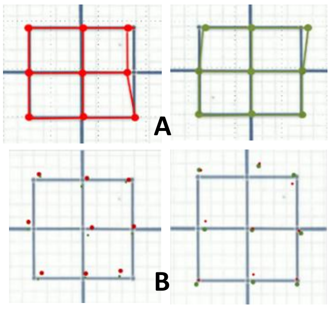

	EXAMINER				EYE-TRACKER			
	Δx LE	Δy LE	Δx RE	Δy RE	Δx LE	Δy LE	Δx RE	Δy RE
1	0.00	0.00	0.00	0.00	1.66	3.98	0.51	1.27
2	0.00	0.00	0.00	0.00	0.33	1.83	0.39	4.97
3	0.00	0.00	-2.50	0.00	-0.58	0.96	0.93	0.05
4	0.00	0.00	0.00	0.00	2.13	4.11	0.40	1.48
5	-2.50	0.00	-3.75	0.00	1.16	1.14	-0.85	-4.33
6	-2.50	0.00	0.00	0.00	0.62	3.62	-1.50	1.24
7	0.00	0.00	0.00	0.00	0.45	2.61	-0.19	2.94
8	0.00	0.00	0.00	0.00	0.33	2.11	-0.72	4.20
9	0.00	0.00	0.00	0.00	1.38	2.97	1.13	2.26

Subject 13

Maddox rod phoria	DV	IV
**Horizontal**	1 Δ esophoria	1 Δ esophoria
**Vertical**	Orthophoria	Orthophoria

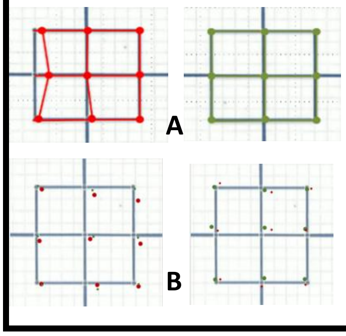

	EXAMINER				EYE-TRACKER			
	Δx LE	Δy LE	Δx RE	Δy RE	Δx LE	Δy LE	Δx RE	Δy RE
1	0.00	0.00	0.00	0.00	-2.04	-1.30	2.78	-0.76
2	5.00	0.00	0.00	0.00	1.02	-2.48	2.10	-1.53
3	2.50	0.00	0.00	0.00	2.44	-1.34	1.91	1.53
4	0.00	0.00	0.00	0.00	1.05	-2.82	2.78	-0.76
5	0.00	0.00	0.00	0.00	2.51	-6.54	1.54	0.47
6	0.00	0.00	0.00	0.00	1.63	-2.88	0.22	-0.36
7	0.00	0.00	0.00	0.00	0.02	-1.28	0.23	-2.58
8	1.25	0.00	0.00	0.00	-0.27	2.09	-1.08	-3.35
9	1.25	0.00	0.00	0.00	0.94	0.55	2.04	0.24

S3.1. Results in Subject 1:

S3.1.1. RE:

HLST measured subjectively or manually: negative horizontal values were found, indicating an exophoria of -5 Δ in primary gaze position (PGP) (point 1), which was maintained when looking to the left (point 2) and to the right. It seemed to decrease slightly at -3.75 Δ (point 6). In the superior gaze at points 3, 4 and 5, a constant exophoria of -5 Δ was maintained, as well as in the downwards gaze (for points 7 and 8), whereas at point 9, it decreased slightly to -2.50 Δ. No vertical deviation was detected at any of the points.

For the measurements obtained objectively with the eye tracker, negative values were also obtained for the PGP (point 1) with an exophoria of -6.08 Δ. In this case, the device measured a higher exophoria when looking to the left of -9.13 Δ (point 2) and a decreased value when looking to the right of -2.62 Δ (point 6). In the upper gaze position (points 3, 4 and 5), exophoria values of -5.48 Δ, -1.03 Δ and -1.03 Δ were obtained, respectively, the last two being very low values, and in the lower gaze position, the value of exophoria increased again at points 7 (-5.80Δ), 8 (-6.60 Δ) and 9 (-8.61).

In contrast, in superior gaze positions, the device measured vertical deviations that were undetected when measured subjectively. In this case, with the measurements made with the eye-tracker, a negative value corresponding to hypophoria of the RE was

collected for the PGP (point 1) of -0.75 Δ, while the left gaze position (point 2) and right gaze position (point 6) had positive values (RE hyperphoria) of 1.22 Δ and 4.64 Δ, respectively. In the superior gaze position (points 3, 4 and 5), RE hyperphoria values of 3.69 Δ, 6.03 Δ and 9.57 Δ, respectively, were obtained, highlighting a greater hyperphoria at point 5 compared to the other points analysed in this direction of gaze. In the lower gaze position, lower RE hyperphoria values at points 7 (1.36 Δ) and 8 (0.20 Δ) were found, while at point 9, a RE hypophoria value of -1.00 Δ was collected.

S3.1.2. LE:

HLST measured subjectively or manually: horizontal orthophoria values (0.00 Δ) were observed, indicating that there was no deviation in PGP (point 1) or in left gaze position (point 2). However, when looking at the right (point 6), an exophoria of -5.00 Δ was observed. In the superior gaze position at point 3, the eye remained in orthophoria, while at points 4 and 5, an exophoria of -2.50 Δ and -5.00 Δ, respectively, was observed. At lower gaze positions, we found values of exophoria at points 7 (-3.75 Δ) and 9 (-6.25 Δ), while at point 8, orthophoria was found again. No vertical deviation was detected at any of the points.

For the measurements obtained objectively with the eye tracker, it was observed that horizontally, there was an exophoria of -4.02 Δ for the PGP (point 1), -4.76 Δ on the left (point 2) and -2.25 Δ on the right (point 6). In the superior gaze position, there was an exophoria of -3.17 Δ at point 3 and -4.23 Δ at point 4, while at point 5, an esophoria of 1.81 Δ was recorded. In the downgaze position, the value of exophoria increased again at points 7 (-5.47 Δ), 8 (-4.78 Δ) and 9 (-7.45 Δ).

The device measured vertical deviations that were not detected manually. Positive values corresponding to hyperphoria of the RE, or its equivalent hypophoria of the LE, of 1.42 Δ at the PGP, 2.08 Δ on the left (point 2) and 3.28 Δ on the right (point 6) were collected. Δ. In the upper gaze position corresponding to points 3, 4 and 5, we found values of 4.30 Δ, 4.32 Δ and 8.57 Δ, respectively, highlighting a greater RE hyperphoria at point 5 compared to the other points analysed in this direction of gaze. In lower gaze positions, we found values of RE hypophoria (or LE hyperphoria) at points 7 (-0.10 Δ) and 9 (-0.15 Δ), while at point 8, a RE hyperphoria of 0.74 Δ was recorded.

S3.1.3. Maddox rod vs. HLST:

When performing the subjective test with the Maddox rod at intermediate vision, Subject 1 had orthophoria horizontally and hypophoria of 1 Δ base-up (BU) RE (-1 Δ) vertically at the PGP. Comparing these subjectively measured results with the Maddox rod with those taken by the eye-tracker in that same gaze position (PGP, point 1), we observed that there was a notable difference in the horizontal component, since the values collected by the eye-tracker were -6.08 Δ in the RE and -4.02 Δ in the LE (both corresponding to an exophoria), but very similar values were obtained vertically, -0.75 Δ in the RE and -0.15 Δ in LE (corresponding to RE hypophoria or LE hyperphoria).

With all the results of this patient, we can determine that he had exophoria in most diagnostic gaze positions not detected with the Maddox rod test but with the HLST in both modalities (manual and with eye-tracker). In addition, in the PGP, the patient presented with RE hypophoria detected by the Maddox rod and eye-tracker but not by the manual HLST. This RE hypophoria became RE hyperphoria in the rest of the diagnostic gaze positions.

S3.2. Results in Subject 3:

S3.2.1. RE:

HLST measured subjectively or manually: positive horizontal values were found indicating an esophoria of 1.25 Δ in PGP (point 1), which was maintained when looking to the right (point 6), and when looking to the left it increased at 2.50 Δ (point 2). In upper

gaze position we find a value of 2.50 Δ at point 3, while points 4 and 5 maintained a constant orthophoria and in lower gaze position, for points 7, 8 and 9 an esophoria value of 2.50 Δ was again found. No vertical deviation was detected at any of the points.

For the measurements obtained objectively with the eye-tracker, positive values were also obtained for the PGP (point 1) with an esophoria of 3.56 Δ. In this case the device had measured a higher esophoria when looking to the left, with 5.46 Δ (point 2), as well as in right gaze position 4.87 Δ (point 6). In superior gaze position (points 3, 4 and 5) values of esophoria were 7.45 Δ, 2.10 Δ and 3.70 Δ respectively, and in inferior gaze positions the esophoria was maintained at points 7 (3.70 Δ), 8 (2.10 Δ) and 9 (3.95 Δ).

In this case the device also measured undetected vertical deviations when measured subjectively. The eye-tracker recorded a negative value corresponding to hypophoria of the RE for the PGP (point 1) of –4.31 Δ, in left gaze position (point 2) a value of -1.76 Δ and changed to a value of 1.07 Δ (RE hyperphoria) to the right (point 6). In superior gaze position (points 3, 4, and 5), the RE hypophoria values were -2.25 Δ, -3.47 Δ, and -3.70 Δ, respectively. In inferior gaze position, we found values of hypophoria in the RE at point 7 (-2.20 Δ), which changed to mild hyperphoria at points 8 (0.40 Δ) and point 9 (2.73Δ).

S3.2.2. LE:

HLST measured subjectively or manually: esophoria of 2.50 Δ appeared in the 9 points examined on the HLST. No vertical deviation is detected at any of the points.

For the measurements obtained objectively with the eye-tracker, we could observe that horizontally there was an esophoria of 1.33 Δ in PGP (point 1), on the left (point 2) of 3.15 Δ and on the right (point 6) of 1.55 Δ. In upper gaze position, there was an esophoria with a higher value at point 3 of 6.25 Δ, 3.54 Δ at point 4 and 5.50 Δ at point 5. In lower gaze position, the esophoria values were at points 7 (2.35 Δ), 8 (3.01 Δ) and 9 (3.47 Δ).

The device measured vertical deviations that were not detected manually. Negative values corresponding to RE hypophoria (-3.67 Δ), or its equivalent LE hyperphoria, were found in PGP and in left gaze position (point 2) of -1.05 Δ. In right gaze position (point 6) a positive corresponding to a RE hypophoria of 0.25 Δ was obtained. In the upper gaze position corresponding to points 3, 4 and 5 we found values of -1.33 Δ, -2.54 Δ and -2.23 Δ respectively. In inferior gaze position, we found values of RE hypophoria (or LE hyperphoria) at points 7 (-1.20 Δ) and 8 (-0.68 Δ), while at point 9 a RE hyperphoria of 1.13 Δ was recorded.

S3.2.3. Maddox rod vs. HLST:

When performing the subjective test with the Maddox rod at intermediate vision, Subject 3 had a horizontal esophoria of 2 Δ and a vertical hyperphoria of 1 Δ base-down (BD) RE (1 Δ) at the PGP. Comparing these subjectively measured results with the Maddox Rod with those taken by the eye-tracker in that same gaze position (point 1), the values were similar in the horizontal component, since the values collected by the eye-tracker were 3, 56 Δ in the RE and 1.33 Δ in the LE (both corresponding to an esophoria). But quite different values were obtained vertically, being -4.31 Δ in the RE and -3.67 Δ in the LE (corresponding to RE hyophoria or LE hyperphoria).

S3.3. Results in Subject 9:

S3.3.1. RE:

HLST measured subjectively or manually: negative horizontal values were found indicating an exophoria of -20.00 Δ in PGP (point 1), which was maintained when looking to the right (point 6), and when looking to the left it decreased at -15.00 Δ (point 2). In upper gaze position a value of -20.00 Δ at the three points 3, 4 and 5 was maintained constant, and in lower gaze position, for point 7 the exophoria increased at –22.50 Δ, for

point 8 was maintained at –20.00 Δ and for point 9 the exophoria value decreased at -15.00 Δ. No vertical deviation was detected at any of the points.

For the measurements obtained objectively with the eye-tracker, negative values were also obtained for the PGP (point 1) with an exophoria of -0.20 Δ. In this case the device had measured a lower exophoria when looking to the left, with –2.60 Δ (point 2), as well as in right gaze position –10.18 Δ (point 6). In superior gaze position (points 3, 4 and 5) values of exophoria were –10.78 Δ, -4.30 Δ and –7.00 Δ respectively, and in inferior gaze positions the exophoria was maintained at points 7 (-8.53 Δ), 8 (-1.20 Δ) and 9 (-1.90 Δ).

In this case the device also measured undetected vertical deviations when measured subjectively. The eye-tracker recorded a positive value corresponding to hyperphoria of the RE for the PGP (point 1) of 3.70 Δ, in left gaze position (point 2) a hypophoria of -6.80 Δ and changed to a value of 10.65 Δ (RE hyperphoria) to the right (point 6). In superior gaze position (points 3, 4, and 5), the RE hyperphoria values were 7.62 Δ, 5.90 Δ, and 10.65 Δ, respectively. In inferior gaze position, we found values of hypophoria in the RE at point 7 (-3.80 Δ), which changed to mild hyperphoria at points 8 (6.60 Δ) and point 9 (8.80Δ).

S3.3.2. LE:

HLST measured subjectively or manually: negative horizontal values were found indicating an exophoria of -5.00 Δ in PGP (point 1), which was increased when looking to the right at –20.00 Δ (point 6), and when looking to the left it increased at -6.25 Δ (point 2). In upper gaze position exophorias of -5.00 Δ, -15.00 Δ and -10.00 Δ were found at points 3, 4 and 5, respectively. In lower gaze position, a value of -15.00 Δ at the three points 7, 8 and 9 was maintained constant. No vertical deviation was detected at any of the points.

For the measurements obtained objectively with the eye-tracker, horizontally there was an almost insignificant exophoria of –0.08 Δ in PGP (point 1), on the left (point 2) of -4.00 Δ and on the right (point 6) of –6.00 Δ. In upper gaze position, there was an exophoria with a higher value at point 3 of -6.68 Δ, -1.03 Δ at point 4 and -5.52 Δ at point 5. In lower gaze position, the exophoria values were at points 7 (-1.73 Δ), 8 (-1.53 Δ) and 9 (-8.45 Δ).

The device measured vertical deviations that were not detected manually. Positive values corresponding to RE hyperphoria (2.13 Δ), or its equivalent LE hypophoria, were found in PGP, in left gaze position (point 2) of 8.97 Δ and in right gaze position (point 6) of 9.25 Δ. In the upper gaze position corresponding to points 3, 4 and 5 we found values of 6.03 Δ, 2.43 Δ and 7.44 Δ respectively. In inferior gaze position, we found values of RE hyperphoria (or LE hypophoria) at points 7 (7.80 Δ), 8 (8.47 Δ), and 9 (4.25 Δ).

S3.3.3. Maddox rod vs. HLST:

When performing the subjective test with the Maddox rod at intermediate vision, Subject 9 had a horizontal exophoria of -1 Δ and vertical orthophoria at the PGP. Comparing these subjectively measured results with the Maddox Rod with those taken by the eye-tracker in that same gaze position (point 1), the values were similar in the horizontal component, since the values collected by the eye-tracker were –0.20 Δ in the RE and –0.08 Δ in the LE (both corresponding to an exophoria). But quite different values were obtained vertically, being 3.70 Δ in the RE and 2.13 Δ in the LE (corresponding to RE hyperphoria or LE hypophoria).
